# Anabolic Steroid Initiation Among Boys and Young Men After Use of Muscle-Building Supplements

**DOI:** 10.1001/jamanetworkopen.2024.50566

**Published:** 2024-12-12

**Authors:** Abigail Bulens, Jerel P. Calzo, Trine Tetlie Eik-Nes, Ariel Beccia, Amanda Raffoul, Vishnudas Sarda, S. Bryn Austin

**Affiliations:** 1 Division of Adolescent and Young Adult Medicine, Boston Children’s Hospital, Boston, Massachusetts; 2 Division of Health Promotion and Behavioral Science, San Diego State University, San Diego, California; 3 Department of Pediatrics, Harvard Medical School, Boston, Massachusetts; 4 Department of Neuroscience and Behavioral Medicine, Norwegian University of Science and Technology, Trondheim, Norway; 5 Stjørdal Community Mental Health Centre, Nord-Trøndelag Hospital Trust, Levanger, Norway; 6 Department of Nutritional Sciences, University of Toronto, Toronto, Ontario, Canada; 7 Department of Social and Behavioral Sciences, Harvard T.H. Chan School of Public Health, Boston, Massachusetts

## Abstract

This cohort study examines the prospective association between muscle-building supplement use among boys and young men and subsequent initiation of anabolic-androgenic steroids.

## Introduction

Social pressure on boys to increase muscularity has intensified over the past several decades,^1^ leading many to use inadequately regulated^2^ muscle-building dietary supplements (MBSs).^1^ MBSs often contain undisclosed pharmaceuticals, illicit steroids, excessive stimulants, and other toxic ingredients^2,3^ and are frequently inaccurately labeled,^4^ sometimes causing serious physical and psychological^5^ consequences and potentially acting as a gateway to anabolic-androgenic steroid (AAS) use.^6^ Our study aimed to estimate the prospective association between MBS use and incident AAS use in boys and young men.

## Methods

We analyzed data from 2 Growing Up Today Study prospective cohorts (GUTS1 and GUTS2), which enrolled more than 27 000 US adolescents in 1996 and 2004.^1^ The Brigham and Women’s Hospital Human Subjects Committee approved this study. Youths provided assent, and caregivers and adult participants provided informed consent. This study followed the STROBE reporting guideline.

Our sample included cisgender boys and young men (aged 10-27 years) who completed self-report surveys annually or biennially over 6 survey waves spanning 14 years (2007-2021). Participants were queried about gender identity, racial identity (Black, White, or other race or ethnicity), and past-year substance use to build muscle (eg, use of protein shakes or powders, creatine, amino acids, hydroxymethylbutyrate, and dehydroepiandrosterone, combined into a binary [yes or no] MBS use variable). Racial identity was included because use differences among racial and ethnic groups have been reported. Past-year prevalent use of AAS to build muscle (yes or no) was also queried. Prevalence of MBS and AAS use reported over the 14 years was estimated.

For subanalyses estimating the prospective association of MBS use with new-onset AAS use, we excluded 4 participants because they reported AAS use at the same wave or the wave prior to responding to the MBS questions and 277 participants because they did not respond to the AAS question subsequent to responding to the MBS questions. AAS use was counted only once in the model estimating incident use. MBS use was assessed 1 wave before AAS use assessment, with the interval between assessments ranging from 1 to 5 years. Multivariable logistic models estimated the odds ratio (OR) and 95% CI for the prospective association between MBS use at a given study wave and initiation of AAS use by the next wave. Models controlled for age and cohort and were fit using generalized estimating equations to account for repeated measures and sibling clusters. We used SAS/STAT, version 15.1 (SAS), for analysis; 2-sided *P* < .05 indicated statistical significance.

## Results

There were 4073 respondents (baseline mean [SD] age, 20.3 [3.7] years) over the 6 survey waves. Among them, 3387 (92.8%) were White and 263 (7.2%) were another race or ethnicity (including American Indian or Alaska Native, Asian or Pacific Islander, Black, Hispanic, and other or unlisted race or ethnicity); 423 (10.4%) did not report their race or ethnicity. In the first wave, 348 respondents (11.1%) reported past-year MBS use and 12 (0.4%) reported AAS use. A total of 1534 respondents (37.7%) ever reported any past-year MBS use and 22 (0.5%) ever reported any past-year AAS use. In the incident-use subanalysis of 3792 participants (9672 responses across 6 survey waves), 2796 responses (28.9%) indicated past-year MBS use (Table 1). More MBS users (14 [0.5%]) reported new-onset AAS use at the next wave compared with non-MBS users (4 [0.1%]). MBS users had more than 8 times the odds of initiating AAS use by the next survey wave (adjusted OR [AOR], 8.31 [95% CI, 2.59-26.73]) compared with non-MBS users (Table 2). Age (AOR, 0.98 [95% CI, 0.85-1.12]) and cohort (AOR, 0.83 [95% CI, 0.30-2.32]) were not statistically significant.

**Table 1.** Baseline Characteristics of Cisgender Boys and Young Men and Past-Year MBS Use Over 6 Survey Waves, 2007-2021

Characteristic	Value (n = 3792 participants and 9672 responses)
**Baseline (2007)**	
Age, mean (SD) [range], y	20.3 (3.7) [10-27]
Cohort, No. of participants (%)	
GUTS1	2427 (64.0)
GUTS2	1365 (36.0)
**MBS use over 6 survey waves (2007-2021)**	
Past-year MBS use, No. of responses (%)	
Yes	2796 (28.9)
No	6876 (71.1)

Abbreviations: GUTS, Growing Up Today Study; MBS, muscle-building supplement.

**Table 2.** Prospective Association of Past-Year MBS Use With Incident Past-Year AAS Use at Next Wave Gathered Across 6 Survey Waves, 2007-2021

Subsequent incident AAS use at next survey wave	No. (%) of responses^a^	AOR (95% CI)
Among MBS users (total responses across 1759 participants = 2796)	14 (0.5)	NA
Among MBS nonusers (total responses across 3024 participants = 6876)	4 (0.06)	8.31 (2.59-26.73)

Abbreviations: AAS, anabolic-androgenic steroid; AOR, adjusted odds ratio; MBS, muscle-building supplement; NA, not applicable.

^a^ Across multiple repeated measures (range, 1-4 responses) from participants (n = 3792 participants and 9672 responses).

## Discussion

In this cohort study, MBS users had substantially elevated odds of incident AAS use within 1 to 5 years. This finding supports the hypothesis of Hildebrandt et al^6^ that MBS use is a gateway for escalating risk-taking behaviors to increase muscularity. One limitation of this study is the wide CI around the OR.

The health risks of MBS use are well documented,^5^ as inadequate federal regulation^4^ has resulted in a US MBS marketplace rife with inaccurate labeling^4^ and adulteration with toxic ingredients.^2,3^ Clinicians, coaches, and parents should counsel against MBS use. Future studies with larger and more diverse samples are needed.

## Notes

Supplement.
Data Sharing Statement
