# Supplementary material for: Anabolic Steroid Initiation Among Boys and Young Men After Use of Muscle-Building Supplements
Source: JAMA Netw Open. 2024 Dec 12;7(12):e2450566. doi: 10.1001/jamanetworkopen.2024.50566 (PMC11638791; doi:10.1001/jamanetworkopen.2024.50566)
Supplement: Supplement. — Data Sharing Statement [file jamanetwopen-e2450566-s001.pdf]

Data Sharing Statement

Bulens. Anabolic Steroid Initiation Among Boys and Young Men After Use of Muscle-Building Supplements. *JAMA Netw Open*. Published December 12, 2024.
doi:10.1001/jamanetworkopen.2024.50566

Data
Data available: Yes
Data types: Other (please specify)
Additional Information: Readers can contact the authors for the data sharing policy of the Channing Division of Network Medicine, which manages the Growing Up Today Study cohort.
How to access data: All individual-level data came from the Growing Up Today Study 1 and 2 (N>27,000), two population-based cohorts of U.S. youth aged 9–15 years at baseline in 1996 and 2004, respectively, who have since been surveyed annually or biannually; further details, including procedures for requesting data access, can be found elsewhere (https://gutsweb.org/collaborate-with-guts/).
When available: With publication

Supporting Documents
Document types: None

Additional Information
Who can access the data: Readers can contact the authors for the data sharing policy of the Channing Division of Network Medicine, which manages the Growing Up Today Study cohort.
Types of analyses: Readers can contact the authors for the data sharing policy of the Channing Division of Network Medicine, which manages the Growing Up Today Study cohort.
Mechanisms of data availability: Readers can contact the authors for the data sharing policy of the Channing Division of Network Medicine, which manages the Growing Up Today Study cohort.
